# Non-solvolytic synthesis of aqueous soluble TiO_2_ nanoparticles and real-time dynamic measurements of the nanoparticle formation

**DOI:** 10.1186/1556-276X-7-297

**Published:** 2012-06-07

**Authors:** Lan Chen, Kamil Rahme, Justin D Holmes, Michael A Morris, Nigel KH Slater

**Affiliations:** 1 Department of Chemical Engineering and Biotechnology, University of Cambridge, Pembroke Street, Cambridge, CB2 3RA, UK; 2 Faculty of Natural and Applied Sciences, Department of Sciences, Notre Dame University (NDU), Louaize, Zouk Mosbeh, Lebanon; 3 Materials Section and Supercritical Centre, Department of Chemistry, University College Cork, Cork, Ireland

**Keywords:** Synthesis, Nanoparticles, TiO_2_, Aqueously soluble, Direct liquid phase precipitation, Dynamic real-time measurement

## Abstract

Highly aqueously dispersible (soluble) TiO_2_ nanoparticles are usually synthesized by a solution-based sol–gel (solvolysis/condensation) process, and no direct precipitation of titania has been reported. This paper proposes a new approach to synthesize stable TiO_2_ nanoparticles by a non-solvolytic method - direct liquid phase precipitation at room temperature. Ligand-capped TiO_2_ nanoparticles are more readily solubilized compared to uncapped TiO_2_ nanoparticles, and these capped materials show distinct optical absorbance/emission behaviors. The influence of ligands, way of reactant feeding, and post-treatment on the shape, size, crystalline structure, and surface chemistry of the TiO_2_ nanoparticles has been thoroughly investigated by the combined use of X-ray diffraction, transmission electron microscopy, UV-visible (UV–vis) spectroscopy, and photoluminescence (PL). It is found that all above variables have significant effects on the size, shape, and dispersivity of the final TiO_2_ nanoparticles. For the first time, real-time UV–vis spectroscopy and PL are used to dynamically detect the formation and growth of TiO_2_ nanoparticles in solution. These real-time measurements show that the precipitation process begins to nucleate after an initial inhibition period of about 1 h, thereafter a particle growth occurs and reaches the maximum point after 2 h. The synthesis reaction is essentially completed after 4 h.

## Background

Titania (TiO_2_) is an important oxide with commercially exploitable physical and chemical properties. There are a variety of applications in, e.g., gas sensing [1-3], catalysis [4,5], photocatalysis [6-8], optics [9-14], photovoltaics [15-17], and pigmentation [18,19]. TiO_2_ nanoparticles (NPs) have been intensively studied over the past two decades. Several different methods have been developed for the synthesis of TiO_2_ NPs, e.g., sol–gel, reverse micelle method [20], and non-hydrolytic method [21]. Among them, the solution-based sol–gel synthetic route is the most widely used approach and consists of two continuous steps: solvolytic reaction (usually hydrolysis) of a titanium salt and a further condensation reaction. Based on the solvent used as the reactant in the first step, it can be described as hydrolysis (reaction with water) [22-25], alcoholysis (reaction with alcohols) [26-28], and ammonolysis (reaction with ammonium ions) [29,30]. The synthesis reaction and subsequent treatments are carried out under ambient [31-33] or solvothermal conditions [11,34-38]. Recently, a novel solution-based method to synthesize nanostructured metal oxides - the direct liquid phase precipitation (DLPP), has been developed by us [39]. In this approach, a great variety of metal oxide NPs can be easily prepared by exchange of anions of a corresponding metal salt (e.g., CuCl, ZnCl_2_, FeCl_3_, and SnCl_4_) with alkali metal oxides in non-aqueous conditions. Here, the method is used to make aqueously soluble TiO_2_ NPs from their titanium salt and lithium oxide in the presence of some amphiphilic capping ligands, i.e., gallic acid and dopamine, where the surface functionalization of the TiO_2_ NPs is made *in situ* (within the synthesis mixture) so as to define NP dimension and dispersivity.

## Methods

### General procedure for bare TiO_2_ NP synthesis

In a typical synthesis (stoichiometrical non-synchronous addition), 0.5 to 1.0 mmol of titanium(IV) chloride tetrahydrofuran complex was dissolved into 10 mL of anhydrous ethanol, forming yellow-like clear solution A. Lithium oxide of 1.0 to 2.0 mmol was dispersed into 10 mL of anhydrous ethanol by sonication for 30 min. This solution was filtered using a PTFE filter membrane (0.45 μm pore size) to form cloudy solution B. Then, solutions A and B were mixed either in a simple way by pouring B into A within 1 s or in a slow way by feeding B into A using a syringe pump within 1 h (non-synchronous) under rigorous stirring at room temperature (RT). After 24 h, a white precipitate was formed upon slow ethanol evaporation, where about two-thirds of ethanol was evaporated in the case of pouring addition or formed directly in the case of syringe pump feeding. Then, the white precipitate was collected by filtration using Nylon filter membranes (0.22 μm pore size) and washed with absolute ethanol three times before drying overnight in air at 60 °C. In order to check the dynamic process of the precipitation reaction, solutions A and B were added at the same time with the same rate (synchronous) or different rate (non-synchronous) using a syringe pump.

For a synchronous addition, 10 mL of solutions A and B was simultaneously and stoichiometrically added to a reactor pre-filled with 20 mL of anhydrous ethanol using a syringe pump (model: KDS–270, KD Scientific Inc., Holliston, MA, USA) at a feeding rate of 2 mL·h^−1^ under rigorous stirring at RT. The resultant mixture was divided into two equal portions. One portion of the solution was constantly stirred at RT under exposure to ambient until a white precipitate was formed. The other portion of the solution was transferred into 30 mL of deionized water in a Teflon-lined autoclave which was then constantly heated at 150 °C for 24 h until a white precipitate was harvested. The white precipitates of both reactions were collected by filtration and washed with absolute ethanol three times before drying overnight in air at 60 °C. The non-synchronous feeding was carried out in a non-stoichiometric way, where 1.0 mL of solution B was transferred to a reactor and the remaining 9 mL of solution B and 10 mL of solution A were then fed synchronously (1 mL·h^−1^) into the reactor under rigorous stirring at RT. The precipitated products were collected by centrifugation at a speed of 10,000 rpm for 20 min and washed with absolute ethanol three times and dried overnight under vacuum at RT.

### Gallic acid-capped TiO_2_ NP synthesis

Gallic acid (GalA) of 0.25 mmol was added into 10 mL of 0.05 M titanium(IV) chloride tetrahydrofuran complex (i.e., solution A above) prior to the addition of 10 mL of 0.1 M lithium oxide (solution B) as described in the general procedure. The resultant mixture was stirred (in ambient as previously described) until a yale precipitate was formed. The precipitate was harvested, washed, and dried as described above.

### Dopamine-capped TiO_2_ NP synthesis

The process is similar to the previous process except that an equivalent mole (0.25 mmol) of dopamine hydrochloride (Dpa) was used instead of gallic acid.

Powder X-ray diffraction (XRD) patterns were recorded on a Philips X’pert MPD diffractometer (Amsterdam, The Netherlands) using Cu Kα radiation and a working voltage of 40 kV. Transmission electron micrographs (TEM) were taken on a JEM-2011 (Jeol Ltd., Akishima-shi, Japan) electron microscope operating at 200 kV. TiO_2_ NPs were dispersed into ethanol before use, and one or two drops of the above solution were transferred onto a holey carbon film on copper grids under dry ambient atmosphere at RT and dried overnight. Photoluminescence (PL) and UV-visible (UV–vis) absorbance spectra were obtained using a PerkinElmer LS50B fluorescence spectrometer (Waltham, MA, USA) and a Cary 50 UV-visible spectrophotometer (Agilent Technologies, Inc., Santa Clara, CA, USA), respectively, where 3 mL of clear aqueous solutions containing TiO_2_ nanoparticles were placed in quartz cuvette cells for the optical analysis. The real-time UV–vis absorbance spectra were collected at intervals of 1 min up to 5 h.

## Results and discussion

**Figure 1:**
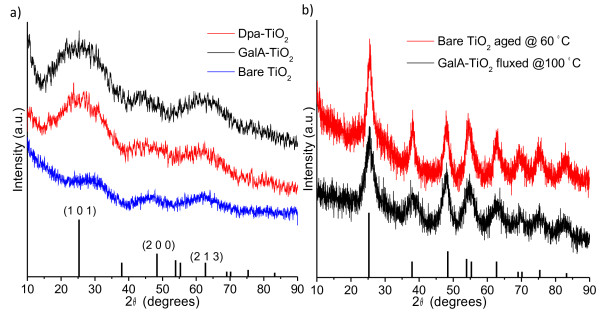
**XRD patterns.** As-synthesized TiO_2_ NPs at RT (**a**) and the TiO_2_ (anatase) NPs aged at elevated temperature (**b**) with the corresponding reference file (ICDD-PDF No. 01–0562).

Figure 1a shows diffractograms for as-synthesized TiO_2_, GalA-, and Dpa-TiO_2_ NP samples. Three poorly resolved diffraction peaks are present for each sample indicating the formation of nearly X-ray-amorphous TiO_2_ clusters in each case. The reflections can be indexed as (1 0 1), (2 0 0), and (2 1 3) plane for a tetragonal (anatase) cell. The crystallite size estimated by Scherrer’s equation is less than 1 nm in all cases. However, enhanced crystallization can be observed in all samples when prepared at higher treatment temperatures, e.g., aged at 60 °C or fluxed at 100 °C overnight. Typical data are shown in Figure 1b, and the samples reveal a series of well-defined X-ray reflections in the 2*θ* range of 10° to 90°. These can be indexed to a tetragonal anatase cell (ICDD-PDF No. 01–0562) with lattice parameters of *a* = 3.73 Å and *c* = 9.37 Å. The crystal sizes are 5.3 and 3.0 nm for the bare and gallic acid-capped samples, respectively, as estimated by Scherrer’s equation (Figure 1b). Both bare and ligand-capped samples show significant Ostwald ripening upon the elevated temperature. A dramatic enhancement in diffraction intensity seen at 60 °C and 100 °C suggests that the as-synthesized TiO_2_ clusters are thermodynamically unstable and are easy to be grown into larger particles upon heating, even very mild heating.

**Figure 2:**
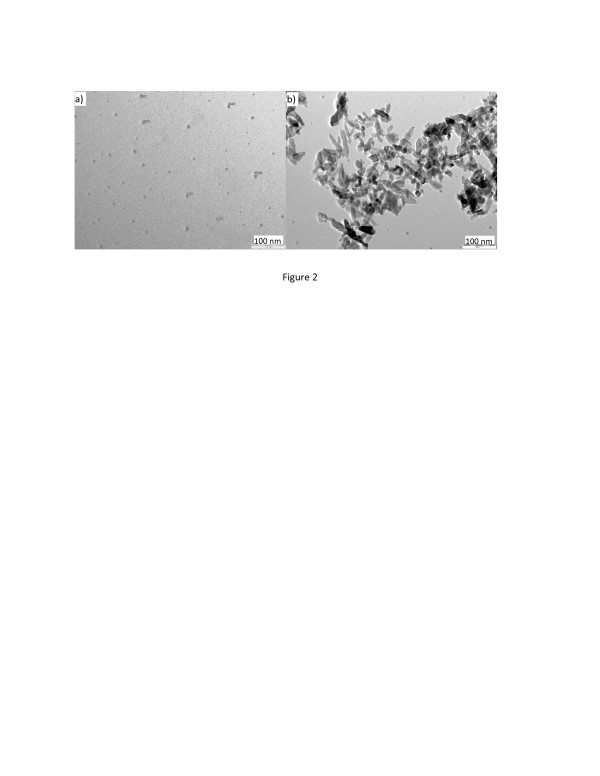
**TEM images of as-synthesized bare TiO**_**2**_**NPs (a) and the hydrothermally treated TiO**_**2**_**NPs (b).** The latter (in a mixture of EtOH/H_2_O = 1:1.5 at 150 °C for 24 h) are from a simple stoichiometric non-synchronous feeding where the equivalent mole of Li_2_O and TiCl_4_ solutions were fed at a molar rate of M_Li2O_/M_TiCl4_ = 2:1.

**Figure 3:**
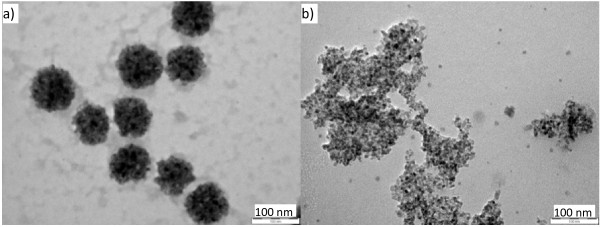
**TEM images of as-synthesized bare TiO**_**2**_**NP aggregates (a) and hydrothermally aged TiO**_**2**_**NPs (b).** The latter (in a mixture of EtOH/H_2_O = 1:1.5 at 150 °C for 24 h) are from a non-stoichiometric synchronous feeding at a molar rate of M_Li2O_/M_TiCl4_ = 1.8:1.

Figure 2a shows that the primary NPs range from 4 to 11 nm and have strong tendency to accumulate to larger aggregates (see Additional file 1: Figure S1). Therefore, the size-polydispersed secondary NP aggregates are always accompanied with the primary NPs in any such preparation. The primary particle size is somewhat greater than the crystallite size observed by XRD and indicates that the particles are largely amorphous in nature. Hydrothermal synthesis at 150 °C for 24 h produces larger TiO_2_ nanostructures in the form of nanorods (NRs) as shown in Figure 2b. The NRs have an aspect ratio of 4 to 5, and the length and width are measured at 70 ± 20 and 15 ± 5 nm, respectively (see Additional file 1: Figure S2). The significant change in morphology after hydrothermal treatment is consistent with the high thermodynamic instability of the primary precipitated product where the larger NP aggregates are dissolved and re-crystallized into the rod-like particles. The *in situ* O^2−^ (Li_2_O)-to-Ti^4+^ (TiCl_4_) ratio used in the synthesis has a significant effect on the precipitation rate, and it was found that the greater the *in situ* O^2−^/Ti^4+^ ratio, the faster the precipitation and the larger the formed particle aggregates are. Variation of the *in situ* O^2−^/Ti^4+^ ratio can be achieved when the synthesis reaction is carried out by a synchronous feeding of a non-stoichiometric mixing of two reactants at a different rate where a small portion of Li_2_O solution, e.g., 10% *v*/*v* of the required volume, is pre-filled in the reactor prior to the feeding. Figure 3a shows the formation of large secondary TiO_2_ particles with an average diameter of 96 nm (see Additional file 1: Figure S2). These large secondary particles are NP aggregates which consist of larger numbers of sub-10-nm-sized primary NPs.

Hydrothermal treatment of these non-stoichiometric preparations produces very different materials from those in Figure 2b. TEM (Figure 3b) shows that the as-synthesized NPs aged under the hydrothermal conditions have almost similar size as those in the simple stoichiometric preparation (Figure 2a). The small-sized NPs tend to aggregate on the TEM grid (Figure 3b) due to the strong binding interaction caused by the large specific surface area. These small sphere-like particles sharply contrast the rod-like product synthesized by the stoichiometric reaction (Figure 2b). The shape difference of the hydrothermally aged stoichiometric and non-stoichiometric samples probably derives the Ostwald ripening to a different extent due to the different particle size distribution. In the stoichiometric preparation, the as-synthesized products are polydisperse particles with size distribution in a quite broader range. The larger particle aggregates have a slower dissolution rate than the smaller particles under hydrothermal conditions and need a longer time to reach the dissolution-recrystallization equilibrium. Therefore, it favors NRs to be grown under this condition. For the non-stoichiometric reaction, the particles have largely uniform/monodisperse NP aggregates (Figure 3a) and the thermodynamic driving force for the Ostwald ripening is, therefore, minimized.

**Figure 4:**
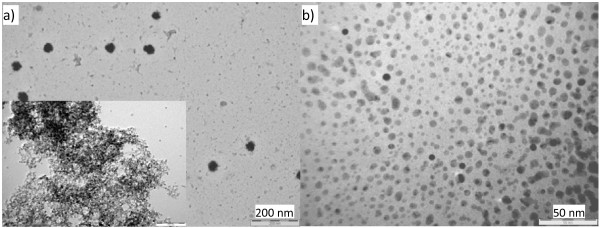
**TEM images of as-synthesized bare TiO**_**2**_**NPs by non-synchronous feeding with different starting Li**_**2**_**O concentrations.** (**a**) 25 mM (inset: the hydrothermally treated sample ‘a’ in a mixture of EtOH/H_2_O = 1 at 150 °C for 24 h) and (**b**) 50 mM.

The NP structure has little change on its primary particle when the reactant solution is fed by the non-synchronous way instead of the synchronous way. Figure 4a shows that the NPs formed in low concentration upon the dropwise feeding is a composite structure which consists of both approximately 6-nm primary NPs and approximately 46-nm secondary NP aggregates (see Additional file 1: Figure S2). However, the secondary NP aggregates are disassembled totally under the hydrothermal treatment and form uniform nanocrystals/NPs of about 4 nm as seen in the inset of Figure 4a (see Additional file 1: Figure S2). The primary NPs are nearly monodispersed when formed in the low concentration, while they become a little broader in their particle size distribution when synthesized in a higher concentration as shown in Figure 4b. The particles have a spherical shape, and the size ranges from 4 to 12 nm with the maximum mode at 6 nm (see Additional file 1: Figure S2). Larger NP aggregates are scarcely formed in the sample obtained in the higher concentration which may be ascribed to a higher concentration of the clusters (nucleus) formed due to the use of a higher initial reactant concentration, and this, therefore, inhibits a fast growth/aggregation for some unstable clusters. The clusters grow up little by little with the continuous reactant feeding and reach to the critical size, where the particles can survive in the solution, in a shorter time under the higher initial concentration than the lower one. In theory, the non-synchronously synthesized NPs should be similar to those mixed by the pouring addition (the simple stoichiometric feeding), but the results show a significant difference. Indeed, the time for the precipitate emergence is obviously shortened from hours to minutes by the non-synchronous feeding than the ‘pouring.’ The synthesis reaction is, in nature, a (ion) precipitation where the *in situ* ion (cluster) environment has significant influence on the kinetics of the NP formation. In other words, the O^2−^/Ti^4+^ ratio is the main driving force for the NP formation. In the non-synchronous feeding process (Figure 4), the O^2−^/Ti^4+^ ratio is highest in the initial stage which highly favors the formation of the nucleus (cluster) and the following NP growth. The ratio becomes lower and lower with the continuous consuming of the O^2−^ species in the reactor and the growth rate of the NPs is lower and lower. The ratio of O^2−^/Ti^4+^ for the simple stoichiometric addition after mixing is almost the same as that of the final stage for the non-synchronous feeding; therefore, the driving force for the NP formation in the former case is significantly lower than the latter one, and the precipitate cannot even be formed in such a sudden mixing (simple stoichiometric addition). So, the solvent evaporation is necessary to accelerate the particle precipitate for this reaction as described above.

### Surface functionalization of TiO_2_ NPs

**Figure 5:**
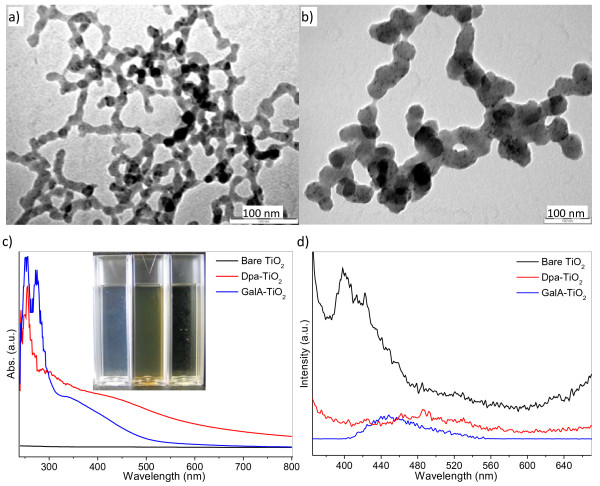
**TEM images, UV–vis absorbance spectra, and PL emission spectra of TiO**_**2**_**NPs.** TEM images of as-synthesized GalA-TiO_2_ NPs (**a**) and Dpa-TiO_2_ NPs (**b**). UV–vis absorbance spectra (**c**) and their photos (inset: from left to right: bare, Dpa- and GalA-TiO_2_ NP aqueous solutions). PL emission spectra (*λ*_*ex.*_ = 350 nm) (**d**) of the NP aqueous dispersions.

More highly water-dispersible (they are sometimes described as ‘soluble’ but, in a strict thermodynamic definition, are not truly soluble) NPs can be obtained by the *in situ* functionalization with some amphiphilic capping ligands, i.e., gallic acid and dopamine chloride. Figure 5a shows the formation of amorphous secondary GalA-TiO_2_ NP chains. The size of these particles is significantly larger than that of the bare NPs due to the presence of the ligand and the formation of the NP aggregates. The Dpa-TiO_2_ NPs also form large secondary particle chains. The primary NP spots can be seen within the secondary particles in Figure 5b. The formation of larger secondary NPs or NP chains for both ligand-capped particles may be contributed to the condensation reaction or hydrogen bonding effect among the functional group like COOH, NH2, and OH on the branch arms on the surfaces of the functionalized NPs. Both gallic acid- and dopamine-capped TiO_2_ NPs have good ‘solubility’ in water and form clear yellow solutions (approximately 10 mg ml^−1^) as shown in Figure 5c. Both NPs are stable in water, and no obvious large aggregates or precipitates can be observed even after several weeks. These dispersions may have a practical value in many applications and are of special interest to us as potential targets to study bio-nano-interaction. The color of Dpa-TiO_2_ is a little darker than that of GalA-TiO_2_ due to the stronger absorbance and scattering ability resulting from the larger size of the Dpa-TiO_2_ aggregates. UV–vis absorbance spectra (Figure 5c) also confirm the solubility difference between the bare TiO_2_ NPs and the ligand-capped TiO_2_ NPs. No significant absorbance lines can be seen in the whole UV–vis spectrum for the bare TiO_2_ system, indicating that the bare TiO_2_ NP solution is quite diluted. In comparison, strong features are present for both ligand-modified NPs. The absorbance intensity of GalA-TiO_2_ NP dispersion in the UV region (<300 nm) is higher than that of Dpa-TiO_2_ while the absorbance in the visible region, especially in the red or the near-infrared spectral region, is weaker than that of Dpa-TiO_2_ which is consistent with the formation of the larger Dpa-TiO_2_ particles as observed in Figure 5b. PL spectra (Figure 5d) show a strong emission peak at 400 nm and several accompanying satellite peaks from 410 to 430 nm for bare TiO_2_ NPs. These data represent a fluorescent blueshift when compared to the values reported by other authors [40,41] which may be attributed to a quantum size effect resulting from the very small nanocrystal size (the cluster - nanocrystal size in bare TiO_2_ NPs, is smaller than 1 nm as estimated by Scherrer’s equation). The presence of the capping ligands results in a photoluminescent ‘quenching’ effect - higher absorbance but low emission ability as seen in Figure 5c,d. A very strong UV–vis absorbance combined with a weak photoluminescent emission indicates that these NPs may have application in solar energy capture or sunscreens.

**Figure 6:**
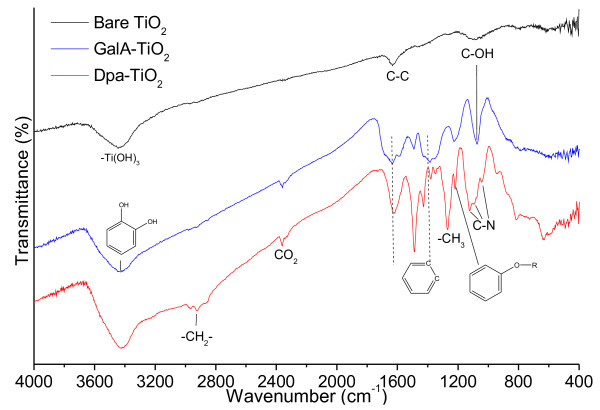
**FT-IR spectra of dried bare Dpa- and GalA-TiO**_**2**_**NPs.**

Fourier transform infrared (FT-IR) data are informative of the surface chemistry of these NPs (Figure 6). The FT-IR peak at 3,440 cm^−1^ indicates the formation of hydroxy groups on the surface of bare TiO_2_ NPs. Peaks at 1,630 (C-C bond) and 1,070 cm^−1^ (hydroxyl-alkyl bond) also imply the absorption of solvent (ethanol) molecules on the bare TiO_2_ NPs. Both GalA-TiO_2_ and Dpa-TiO_2_ NPs have a strong phenol (hydroxy-C bond with benzene ring) signal at 3,420 cm^−1^ and a set of multiple broad (C-C bonds) peaks between 1,640 and 1,400 cm^−1^ corresponding to the trihydroxy- or dihydroxy-benzene unit in gallic acid and dopamine molecule, respectively. However, the Dpa-TiO_2_ NPs can be differentiated from the GalA-TiO_2_ NPs by a strong peak at 2,925 cm^−1^ (C-H bond in methylene group) and the broad peaks from 1,220 to 1,020 cm^−1^ (C-N bonds in the aliphatic amine). The existence of the absorbance peak at 1,220 cm^−1^ indicates the formation of a titanic acid hydroxybenzene ester ((NP)-Ti-O-R, R = benzoic acid or phenethylamine) due to the condensation reaction between TiO_1.5_(OH) and hydroxyl units in gallic acid or dopamine molecule.

### Formation mechanism of TiO_2_ NPs

The DLPP method used here has several advantages in terms of a direct method of synthesizing nanoparticles in a highly controlled environment. A reaction mechanism can be proposed as described below.

Complex formation

(1) $${\text{TiCl}}_{4}\cdot 2\text{THF}+6{\text{CH}}_{3}{\text{CH}}_{2}\text{OH}\overset{}{↔}{\frac{{\left[\text{Ti(THF}\right)}_{2}{{(\text{CH}}_{3}{\text{CH}}_{2}\text{OH})}_{4}]}{\text{complex}}}^{4+}+4{\text{Cl}}^{-}$$

Oxide cluster formation

(2) $${{\left[\text{Ti(THF}\right)}_{2}{{(\text{CH}}_{3}{\text{CH}}_{2}\text{OH})}_{4}]}^{4\text{+}}{\text{+2Li}}_{2}\text{O}\overset{}{↔}\frac{{\text{TiO}}_{2}{\left(\text{THF}\right)}_{2}}{\text{cluster}}{\text{+4Li}}^{\text{+}}{\text{+4CH}}_{3}{\text{CH}}_{2}\text{OH}$$

Nanoparticle nucleation and growth

(3) $$m{\text{ TiO}}_{2}{\left(\text{THF}\right)}_{2}\overset{}{↔}\frac{{{(\text{TiO}}_{2})}_{m}{\left(\text{THF}\right)}_{n}}{\text{nanoparticle}}↓+2m-n\text{ THF}↑(n<<m)$$

As seen in the above equations, TiCl_4_·2THF molecule dissociates and reacts with ethanol to form a new complex (1). This complex ion further reacts with alkali metal oxide (Li_2_O) to form TiO_2_(THF)_2_ cluster which is an unstable oxide precursor (2). Through cluster condensation reaction, the small TiO_2_(THF)_2_ clusters will grow up. The particle growth and precipitation do not occur unless the cluster size reaches to a critical point (3). It is clearly seen from Equation 2 that either the *in situ* increase of the Li_2_O concentration, for example by pouring addition, or the evaporation of ethanol can result in a rapid formation of the clusters (nucleus) and precipitation. However, the solvent evaporation also favors the formation of the final product as depicted in Equation 3. These are consistent with the facts that a non-stoichiometrical addition of TiCl_4_·2THF to Li_2_O solution results in a faster particle precipitation than the synchronous stoichiometrical mixing of the two solutions as shown in Figures 3 and 4.

### Real-time dynamic measurements of the TiO_2_ NP formation

**Figure 7:**
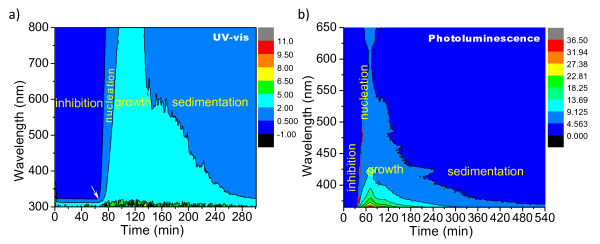
**Real-time UV–vis absorbance (a) and PL emission (*λ***_***ex***_ **= 350 nm) spectra (b) for TiO**_**2**_**NP precipitation.**

For the first time, real-time (RT) measurements of absorbance and emission spectrometry have been used to detect TiO_2_ NP precipitation. They are achieved by the three-dimensional (3D) re-construction of a series of two-dimensional RT UV–vis absorbance and PL emission spectra as shown in Figure 7. Four distinct zones named as ‘inhibition,’ ‘nucleation,’ ‘growth,’ and ‘sedimentation’ can be separated by three clear isoline boundaries in the RT UV–vis spectra in Figure 7a. We only focus on the nucleation and growth stages here since the inhibition and sedimentation stages have less influence on the formation of the TiO_2_ NPs. The re-built 3D plots show how the laser wavelength (*λ*) and the reaction time (*t*) have influenced the absorbance or emission intensity (*I*). The *I*-*λ*-*t* contour map contains two distinct relationships: *I*-*λ* and *I*-*t* . The *I*-*λ* plot is similar to normal UV–vis/PL spectrum lines while the *I*-*t* plot indicates the change of the concentration of the absorbance/emission units in the analyzed space with the reaction time. For these characterizations, the scan starts as soon as two reagent solutions are mixed together. The *I*-*λ*-*t* contour, i.e., the distribution of the change of the concentration (proportional to the intensity) of the product with the time and wavelength, denotes the increase of the number and size of the precipitated particle in the analyzed space. The increase of the particle (cluster) number occurs in the nucleation stage while the increase of the particle size emerges in the growth stage. Both the increase on the size and number of the clusters result in the increase of the absorbance/emission intensity, but the size increase eventually overwhelms the number increase in the particle growth stage while the number increase contributes more intensity to the photon absorbance/emission compared with the size increase in the nucleation stage. It is suggested that quite a few thermodynamically unstable clusters are formed in the first inhibition stage, and these clusters undergo fast dissolution-precipitation equilibrium. Some clusters survive in this time when their size reaches to a critical size and then become stable enough as ‘seeds (nucleuses)’ in the second (nucleation) stage. The RT UV–vis absorbance spectra show that the stable nucleuses with a number that can maintain their self-propagation are not produced during the 64th minute of the inhibition period (as shown by the arrow in Figure 7a). Within a few minutes, the nucleus number suddenly increases following a long inhibition period until the nanosized particle growth (precipitation) becomes inevitable after the short nucleation stage. A following particle growth spontaneously occurs when the nucleuses with enough number exceed the critical size and the growth maintains for a few hours. Finally, the formed NPs/aggregates settle down little by little, and the solution becomes clearer and clearer from top to bottom in the sedimentation stage. It needs to point out that the RT UV–vis analysis is carried out statically without stirring and the nucleus inhibition period is, therefore, a bit longer than that processed with stirring. It is usually hard to differentiate a nucleation and a growth stage by a single normal UV–vis absorbance spectrum (*I*-*λ*) or a single fixed-wavelength dynamic scan (*I*-*t*) because both the nucleation and growth are, in fact, concurrent and cannot be clearly separated either in time (*t*) or in space (*λ*). However, this new measurement method reported here can achieve this by an efficient and intuitionistic way. As we know that TiO_2_ NPs are famous for their strong UV absorbing (for UV screen) and strong visible light reflecting (for white pigment) capabilities, their ‘absorbance’ in the visible light range (>400 nm) are exclusively contributed by the photon ‘blocking’ effect where the emitted photons are scattered rather than absorbed by the NPs along the incident beam direction. As a result, the photon blocking is equal to the photon absorbing on this regard where the blocked (absorbed) photon number is related to both the number and size of the TiO_2_ NPs. It can be confirmed by the isoline boundary line between the nucleation and the growth zone which ascends linearly with the increase of the wavelength and the reaction time. A slope along the boundary line defines the time from the start (67th minute) to the most intense point (97th minute) during the particle growth. On the 97th minute, all spectral maximum absorbance is reached, as the solution changes completely turbid. The maximum absorbance caused by the NP growth maintains for 37 min as shown in Figure 7a. On the 134th minute, the particle growth stops and the formation of the polydisperse particle aggregates starts to dominate the process when all available solutes or the unstable clusters in the solution are completely consumed. A totally clear solution is obtained in the end after 4 h of reaction, when all TiO_2_ NP sediments are in the bottom of the cuvette.

3D PL spectra provide some complimentary information (Figure 7b) on the range where the 3D UV–vis spectra do not work effectively. The 3D emission data show that the nucleation starts on the 33rd minute and reaches the most intense point on the 79th minute which is significantly earlier than that of the corresponding 3D UV–vis absorbance data (67th and 97th minutes). The shorter inhibition time detected in 3D PL emission indicates that large numbers of unstable clusters formed in the inhibition and/or nucleation stage might contribute more to the fluorescence emission rather than the UV–vis absorbance since these tiny clusters show almost no absorbance in the UV–vis spectra (Figure 7a) but stronger emission in the PL spectra (Figure 7b) from the 33rd to the 67th minute. The emission photon wavelength redshift along the emission intensity vs. reaction time isoline is consistent with the particle growth tendency. This agreement might be explained by the understanding on the ‘quantum size effect’ observed in small particles. Larger particles have smaller specific surface area and less strain imposed by the external stress compared with smaller particles. The small particles with higher surface stresses are manifested as a widened energy level gap. Thus, for a given energy level, the emitted photon wavelength (energy) caused by a fixed excitation wavelength, e.g., 350 nm, varies with particle size. The bigger the particle, the longer the wavelength of the emitted photons is, and it is, therefore, reasonable to conclude that the increase of the wavelength of the emitted photon with reaction time is a reflection of the particle growth tendency.

## Conclusions

Aqueously soluble sub-10-nm TiO_2_ NPs have been successfully synthesized by a non-solvolytic method at room temperature. The as-synthesized TiO_2_ NPs are largely amorphous but can be crystallized by mildly higher temperature or hydrothermal treatment. However, the hydrothermally treated sample shows different morphologies where instead of spherical NPs, a higher aspect ratio product - nanorods - are formed. Both gallic acid- and dopamine-capped TiO_2_ NPs have excellent solubility and stability in water (or other polar solvents) and show distinct UV–vis absorbance and photoluminescent emission properties compared with the uncapped TiO_2_ NPs in aqueous solution. The dispersivity of the TiO_2_ NPs prepared in the presence of gallic acid or dopamine is improved dramatically, and both the carboxylic (GalA)- and amine (Dpa)-capped TiO_2_ NPs tend to form larger secondary particles and have better monodispersivity on them. The different surface chemistries for bare, GalA-, and Dpa-TiO_2_ NPs are clearly revealed by the FT-IR vibrating/stretching features. By varying the feeding procedure, the influence of the (non-) stoichiometric chemistry has been investigated. The stoichiometric feeding favors the formation of polydispersed TiO_2_ NPs while the non-stoichiometric feeding prefers the formation of uniform NP aggregates.

3D real-time measurements show abundant information on the precipitation of TiO_2_ NPs, where a series of progressive reactions involving inhibition, nucleation, growth, and sedimentation have been investigated. Free Ti^4+^ ions react with the alkaline oxide initially to form unstable and stable clusters in the inhibition and the nucleation stage; eventually, thermodynamically stable NPs are formed and settled down in the growth and the sedimentation stage. Both 3D UV–vis and PL spectra confirm a linear growth tendency with reaction time for TiO_2_ NPs.

## Competing interests

The authors declare that they have no competing interests.

## Authors’ contributions

LC carried out the experiments and drafted the manuscript. KR did some of the experiments. JDH gave some good suggestions. MAM and NKHS read the manuscript. All authors read and approved the final manuscript.

## Authors’ information

LC (Ph.D.) is a Marie-Curie Intra-European Fellow. NKHS (Ph.D.) is a professor at the Department of Chemical Engineering and Biotechnology, University of Cambridge, Cambridge, UK. KR (Ph.D.) is an associate professor at the Faculty of Natural and Applied Sciences, Department of Sciences, Notre Dame University, Louaize, Lebanon. MAM (Ph.D.) and JDH (Ph.D.) are professors at the Materials Section and Supercritical Centre, Department of Chemistry, University College Cork, Cork, Ireland.

## Supplementary Material

Additional file 1 **Supporting information.** Particle size distributions for the sample shown in Figures 2a, 3a, 4a, and 4b (Figure S1) and the size of the TiO_2_ nanorods formed by hydrothermal treatment (Figure S2). Click here for file
