# Supplementary material for: Non-solvolytic synthesis of aqueous soluble TiO2 nanoparticles and real-time dynamic measurements of the nanoparticle formation
Source: Nanoscale Res Lett. 2012 Jun 7;7(1):297. doi: 10.1186/1556-276X-7-297 (PMC3461452; doi:10.1186/1556-276X-7-297)
Supplement: Additional file 1 — Supporting information. Particle size distributions for the sample shown in Figures 2a, 3a, 4a, and 4b (Figure S1) and the size of the TiO2 nanorods formed by hydrothermal treatment (Figure S2). [file 1556-276X-7-297-S1.doc]

**Supporting Information**

Figure S1 Particle size distributions for the sample shown in Figure 2a, 3a, 4a and 4b respectively.


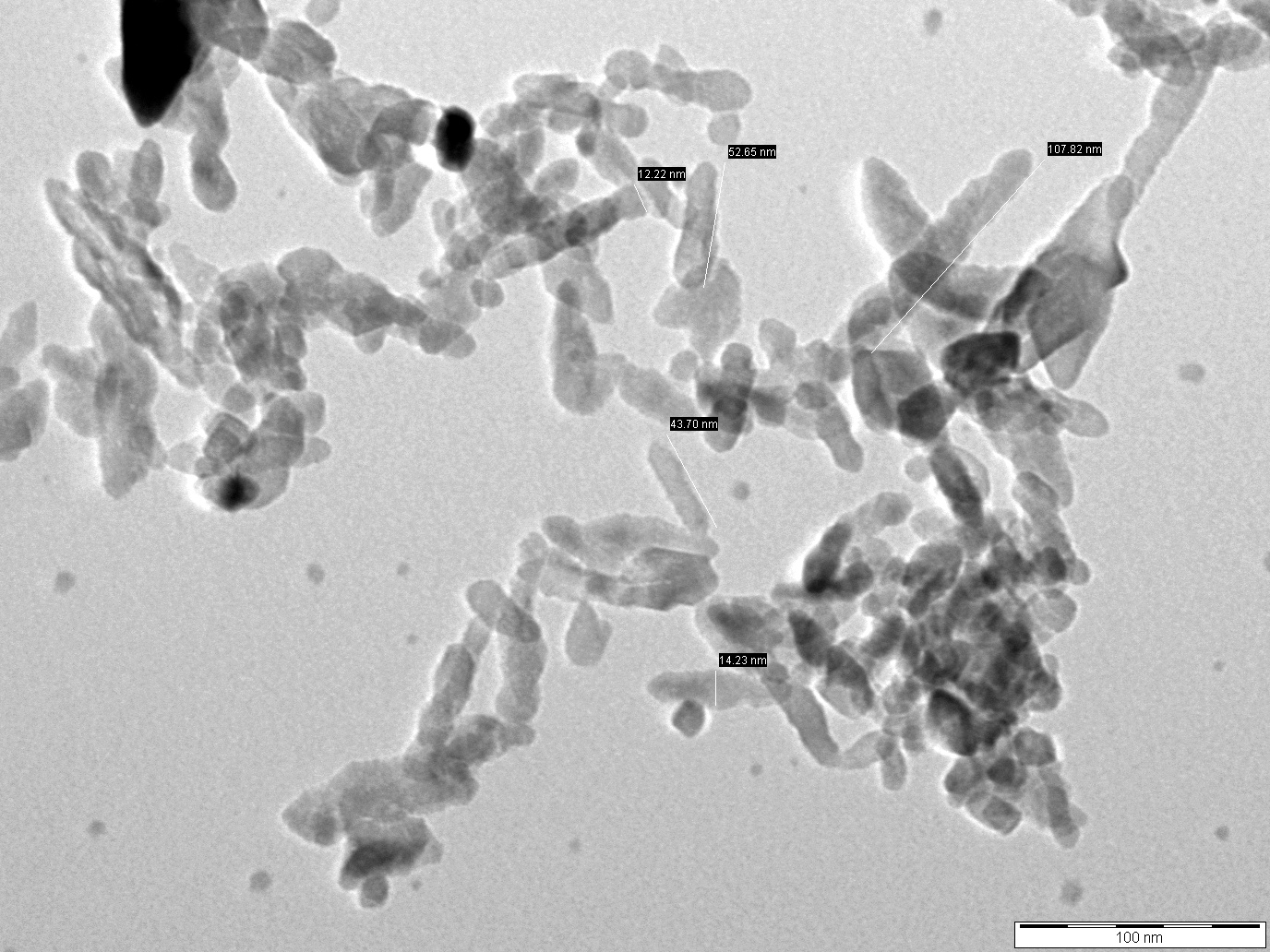


Figure S2 the size of the TiO_2_ nanorods formed by hydrothermal treatment.
